# The impact of educational attainment, intelligence and intellectual disability on schizophrenia: a Swedish population-based register and genetic study

**DOI:** 10.1038/s41380-022-01500-2

**Published:** 2022-04-05

**Authors:** Jie Song, Shuyang Yao, Kaarina Kowalec, Yi Lu, Amir Sariaslan, Jin P. Szatkiewicz, Henrik Larsson, Paul Lichtenstein, Christina M. Hultman, Patrick F. Sullivan

**Affiliations:** 1 Department of Medical Epidemiology and Biostatistics, Karolinska Institutet, Stockholm, Sweden; 2 College of Pharmacy, University of Manitoba, Winnipeg, MB Canada; 3 Department of Psychiatry, University of Oxford, Warneford Hospital, Oxford, UK; 4 Departments of Genetics and Psychiatry, University of North Carolina, Chapel Hill, NC USA; 5 School of Medical Sciences, Örebo University, Örebo, Sweden; 6 Icahn School of Medicine, Department of Psychiatry, Mt Sinai Hospital, New York, NY USA

**Keywords:** Schizophrenia, Genetics

## Abstract

Schizophrenia (SCZ) is highly heterogenous and no subtypes characterizing treatment response or longitudinal course well. Cognitive impairment is a core clinical feature of SCZ and a determinant of poorer outcome. Genetic overlap between SCZ and cognitive traits is complex, with limited studies of comprehensive epidemiological and genomic evidence. To examine the relation between SCZ and three cognitive traits, educational attainment (EDU), premorbid cognitive ability, and intellectual disability (ID), we used two Swedish samples: a national cohort (14,230 SCZ cases and 3,816,264 controls) and a subsample with comprehensive genetic data (4992 cases and 6009 controls). Population-based analyses confirmed worse cognition as a risk factor for SCZ, and the pedigree and SNP-based genetic correlations were comparable. In the genotyped cases, those with high EDU and premorbid cognitive ability tended to have higher polygenetic risk scores (PRS) of EDU and intelligence and fewer rare exonic variants. Finally, by applying an empirical clustering method, we dissected SCZ cases into four replicable subgroups characterized by EDU and ID. In particular, the subgroup with higher EDU in the national cohort had fewer adverse outcomes including long hospitalization and death. In the genotyped subsample, this subgroup had higher PRS of EDU and no excess of rare genetic burdens than controls. In conclusion, we found extensive evidence of a robust relation between cognitive traits and SCZ, underscoring the importance of cognition in dissecting the heterogeneity of SCZ.

## Introduction

Schizophrenia (SCZ) is an often devastating psychiatric disorder associated with substantially elevated rates of impaired social functioning, morbidity, premature mortality, and personal and societal costs [1–4]. SCZ aggregates in families with a sibling recurrence risk ratio of 8.6 which is primarily due to shared genetic influences (twin/pedigree heritability 0.60–0.80) [5–8]. Approximately a third of the twin/pedigree heritability can be attributed to common single-nucleotide polymorphisms (SNP-heritability 0.24) [9, 10]. It is now firmly established that both common and rare genetic variation influence the risk of SCZ, as in most other complex diseases [11–15].

Impaired cognitive ability is an important clinical feature of SCZ [16] and a determinant of poorer outcome [17]. Lower premorbid cognitive ability is a risk factor for SCZ [18] and cognitive ability can decline after SCZ onset [19]. Intellectual disability (ID) is defined by marked impairment in cognitive ability and is an important comorbidity of SCZ [20].

The genetic relationship between cognitive ability and SCZ is complex. Common genetic variants contribute to both cognitive traits and rare severe neurodevelopmental disorders including ID. Recent studies have found shared loci between intelligence and SCZ along with a negative SNP-based genetic correlation (*r*_g_ = −0.21) and Mendelian randomization analyses suggested bidirectional causal effects [21, 22]. Previous studies reported a positive genetic correlation between SCZ and EDU that was attributed to the genetic overlap between SCZ and bipolar disorder (BIP) [23, 24]. However, this correlation was zero by the most recent GWAS [9, 25].

Genetic overlap between SCZ and ID for rare genetic variants of strong effect also exists. Rare predicted loss-of-function (pLoF) exonic variants in *SETD1A* are associated with SCZ and developmental/cognitive delay [26]. Recent research also suggests the associations between pLoF variants (particularly in brain expressed genes) and SCZ [12] as well as educational attainment (EDU) [27]. The cumulative burden of rare pLoF variants is enriched in SCZ cases with comorbid ID, and can predict SCZ risk in individuals without ID [28]. Moreover, unaffected carriers of rare neuropsychiatric copy number variants (CNVs) had cognitive ability intermediate between controls and CNV carriers with SCZ [29].

Given that the extant data strongly hint at important interrelations between genetic risk for SCZ and cognitive traits, we investigated whether cognitive ability and ID might usefully index the etiological or phenotypic heterogeneity of SCZ. We did this by studying two samples, one based on an entire country and second, using a large subset of that country with comprehensive genomic data. First, we evaluated the associations between SCZ, ID, and measures of cognition (EDU and premorbid cognitive ability) using Swedish national register data. Second, we estimated their heritabilities and genetic correlations via a Swedish national sibling cohort. Third, in the genotyped subsample, we assessed whether common and rare genetic variant burden measures (polygenetic risk scores or PRS, CNV burden, and rare exonic burden) usefully added to the results from the national sample [7, 12, 30–33]. Finally, we applied empirical clustering methods to cognitive-related factors to identify SCZ subgroups. To our knowledge, no prior report has considered the relation of cognitive ability, ID, and SCZ while incorporating multiple measures of common and rare genetic variation.

## Methods

### Swedish National Sample

Statistics Sweden maintains national registries containing health service use and governmental data. Unique person numbers (assigned to all Swedish residents at birth or upon immigration [30]) allow linkage of individual data between registers. We were granted access to de-identified data after approval by an Ethical Committee at Karolinska Institutet. We established a national sample of SCZ cases defined as: (a) ≥2 inpatient hospitalizations or specialist outpatient visits with a diagnosis of SCZ or schizoaffective disorder from the National Patient Register; (b) born in Sweden from 1 January 1958 to 31 December 1993 (rationale is that there are incomplete data on older subjects and as we wanted subjects to have entered the core risk period for SCZ by the end of follow-up in 31 December 2013); and (c) excluded individuals with a plausible alternative primary diagnosis (Table S1). This definition of SCZ has been validated widely using clinical, epidemiological, and genetic analyses [6, 7, 11, 32]. We included demographic factors from linkage with other national registers (Supplementary Methods).

### Genotyped subsample from the Swedish SCZ Study (S3)

The S3 genotyped subsample is a subset of the national sample. Full descriptions are in other papers [11, 12, 32–34]. Briefly, blood-derived DNA samples from SCZ cases and controls were collected from 2005–2013. Cases were defined as in the national sample. Controls were selected at random from Swedish population registers and were never hospitalized for SCZ, schizoaffective disorder, or BIP and age ≥ 18 years. S3 was linked to Swedish registers, leaving 4992 cases and 6009 controls with validated status. Due to regulatory prohibitions, we could not remove S3 subjects from the de-identified national sample. All subjects provided informed consent and all procedures were approved by the relevant ethical committees.

### Cognition measures

EDU was derived from a national database coding the highest completed educational level [35]. We coded EDU according to the International Standard Classification of Education as in large Genome-wide association studies (GWAS) [25]. We standardized EDU with respect to birth year and sex into a Z-score. Premorbid cognitive ability, measured as premorbid intelligence quotient (IQ) scores, was obtained from the Conscription Register covering males aged 18–19 from 1967–2010. Individuals with a diagnosis of SCZ at this examination (144 in the national sample and 28 in S3) were excluded from the analyses for premorbid cognitive ability. IQ was Z-score standardized by birth year. ID was defined by medical records using the National Patient Register (Table S1).

### Common and rare measures of genetic burden

Details of genome-wide SNP genotyping, PRS calculation, CNV assessment, and exome sequencing are in the Supplementary Methods. [11, 12, 32–34, 36] Training sets for PRS common variant burden were from the latest GWAS for SCZ, BIP, IQ, and EDU (after removing any Swedish samples) [10, 22, 25, 37]. Rare CNVs are defined by frequency < 0.01, size ≥ 100 kb, and spanning ≥ 15 probes. CNV burden, for duplications and deletions separately, was computed as CNV size (total KB affected by CNVs), total number of CNVs, and the number of pathogenic CNVs (>50% overlap) associated with SCZ, autism, ID, or developmental delay [29, 38–40]. Rare exonic burden was the number of ultra-rare disruptive/damaging single nucleotide variations and indels not observed in Exome Aggregation Consortium study and in constrained genes (previously identified as ‘missense-constrained’ or ‘loss-of-function intolerant’) (Supplementary Methods) [12, 34]. In total, 4288 cases and 5305 controls had available data on all genetic profiles. Genetic burden measures were standardized to aid in interpretation.

### Statistical analyses

We used the national sample to examine associations of SCZ with cognitive traits (i.e., EDU, premorbid cognitive ability and ID) via epidemiological and genetic epidemiological analyses. To assess the impact of the measures of cognitive ability on SCZ risk in the national sample, we fitted Cox regression models that accounted for time at risk. Subjects entered at 1-Jan-1973 and were followed to the date of emigration, death, or up to 31-Dec-2013. First, we examined associations between each cognitive measure and SCZ. Second, we examined the associations of SCZ with EDU and ID jointly. Finally, we examined the associations of SCZ with premorbid cognitive ability, EDU and ID jointly (males only). Relevant epidemiological covariates were adjusted in all models, including sex, birth year, parental EDU, parental age at birth, and whether the person was born in winter.

The national sample can be connected into pedigrees to enable population genetic epidemiological analyses through linkage of Multi-Generation Register. Using an extended twin-family design [41–44], we estimated pedigree heritability by fitting univariate quantitative genetic structural equation models (SEM) separately for SCZ, premorbid cognitive ability, EDU, and ID and decomposing phenotypic variance into additive genetic, shared environmental, and unique environmental components (Supplementary Methods). Sex and birth year were included as covariates to adjust for any group differences. We fitted bivariate quantitative genetic SEM to estimate the pedigree genetic correlations (r_g_) for SCZ with cognitive traits.

We examined the effects of genetic variant burden measures (PRS with *P*_*T*_ ≤ 0.05, CNV and rare exonic burden) on EDU and premorbid cognitive ability. PRS of BIP was tested as previous studies have suggested a positive genome-wide correlation between BIP and EDU [45] and a SCZ subtype resembling BIP and high IQ [23]. Separate models for each genetic burden were evaluated and interaction terms were added to examine whether the effects differed between SCZ cases and controls. Those showed significant associations were then included in a joint model. All statistical models were adjusted for ancestry principal components and genotyping waves.

### Cluster analyses in SCZ cases

Regression methods may not detect the existence of natural groups of patients. Clinicians naturally seek categorical ways to understand patients, and empirical subtyping patients is of intense interest for “patient stratification” to optimize therapeutics. We applied unsupervised clustering to identify subgroups in the national sample. The input variables were cognition-related: EDU, parental EDU, ID, age at first SCZ diagnosis, and the number of BIP hospital contacts [23]. Except for ID, we regressed out birth year and sex for other input variables. As they were nominal and continuous variables, we used Gower’s dissimilarity matrices as input [46]. We used the Uniform Manifold Approximation and Projection (UMAP) to project the embedding of the input matrix into a two-dimensional layout [47]. UMAP is a non-linear dimensional reduction algorithm that preserves data features in lower dimensions and is an effective feature extraction tool in various fields in life science, including population genetics and scRNA-seq [48–50]. We then applied the Density Based Spatial Clustering of Applications with Noise (DBSCAN) to identify clusters [51]. DBSCAN identifies clusters of arbitrary shape and handles outliers more effectively compared to other clustering methods. Clustering replication was evaluated within the national sample via a random 1:1 split into training and replication sets. We applied the same clustering procedures in both training data and replication data and evaluated cluster similarity. After confirming the similarity of the clustering results, we combined the training and replication sets, and fit Cox regression models to compare rates of adverse outcomes in the clusters. Treatment resistance (ever use of clozapine) was shown in proportions but not tested for rates since the data was only available from 2005. Tested adverse outcomes included suicidality (attempts and completed suicide, Table S1), first hospitalization > 200 days (i.e., the median length of hospitalization for those in top decile of hospitalization), and death. SCZ cases were followed from initial SCZ diagnosis to the date of emigration, death, or 31-Dec-2013. Relevant covariates were adjusted for each outcome (Table S5).

Finally, we applied the same clustering procedures in S3 and evaluated the cluster similarity in this genetic subsample. We further examined whether common and rare genetic burdens differ across clusters and from controls (CNV duplications were not tested due to the null association with cognitive measures in previous analyses). Default parameters were used for UMAP algorithm except for specifying *n_neighbors* = 50 and a seed for random number generation (*random_state)* for reproducibility. Parameters for DBSCAN were set to *eps* = 1 and *MinPts* = 50.

### Software and multiple testing corrections

All analyses were performed in R (v4.0.3) [52]. The quantitative genetic models for pedigree analyses were fitted with *OpenMx* (v2.18.1) [53]. Cluster analyses used R packages *cluster*, *umap*, and *fpc*. We performed multiple testing correction with Bonferroni method, which is a conservative correction and works in the worst-case scenario that all tests are independent. Correction on the total number of tests in the study would be overly rigorous and inappropriate. Therefore, we performed Bonferroni correction for groups of related statistical tests rather than the total number of tests performed across the study. This approach is appropriate here (and often found in the psychiatric genomics literature) given that these are distinctive sets of hypotheses and different from running the same analysis on data subsets (e.g., a GWAS for all subjects and then by sex). Here, sets of related statistical tests usually corresponded to the results in a table. The significance thresholds were Bonferroni-corrected to *P* < 0.05, and are given in table legends. Statistical tests were two-sided except for the comparison of SCZ-PRS, CNV, and rare exonic burden between SCZ cases in each cluster and controls, which were one-sided assuming higher rate in cases.

## Results

The Swedish national sample consisted of 14,230 SCZ cases and 3,816,264 controls. The lifetime prevalence of SCZ was 0.37% (95% CI 0.37–0.38%, similar to our 2006 report [6]). Table 1 shows demographic variables in population-level and in the S3 subsample with genetic data. Individuals in S3 were relatively old at recruitment. Both national and genetic sample had profound case-control differences in premorbid cognitive ability, EDU and ID.

**Table 1:** Demographic characteristics of the national sample and genotyped subsample.

*Swedish national sample*	*Cases*	*Controls*	*Statistical comparison*
Subjects	14,230	3,816,264	NA
Birth year, mean (SD)	1969 (8.6)	1975 (10.5)	*t*_3,830,492_ = 72.3, *P* < 1 × 10^−300^
Male sex, *N* (%)	8,762 (61.6%)	1,959,215 (51.3%)	$\chi _1^2$ = 594.2, *P* = 3.12 × 10^−131^
Premorbid cognitive ability (males), mean (SD)	−0.51 (1.07)	0.00 (1.00)	*t*_1,422,976_ = 40.2, *P* < 1 × 10^−300^
Educational attainment, mean (SD)	−0.59 (0.90)	0.00 (1.00)	*t*_3,706,109_ = 69.5, *P* < 1 × 10^−300^
Intellectual disability, *N* (%)	923 (6.5%)	23,692 (0.6%)	$\chi _1^2$ = 7630.1, *P* < 1 × 10^−300^
***Genotyped subsample***	***Cases***	***Controls***	***Statistical comparison***
Subjects	4,992	6,009	NA
Birth year, mean (SD)	1954 (11.8)	1952 (11.3)	*t*_10,999_ = −9.7, *P* = 2.72 × 10^−22^
Male sex, *N* (%)	3,021 (60.5%)	3,052 (50.8%)	$\chi _1^2$ = 103.9, *P* = 2.11 × 10^−24^
Premorbid cognitive ability (males), mean (SD)	−0.34 (0.97)	0.31 (0.91)	*t*_2,544_ = 17.4, *P* = 2.60 × 10^−64^
Educational attainment, mean (SD)	−0.34 (0.87)	0.28 (1.00)	*t*_10,770_ = 33.7, *P* = 2.18 × 10^−237^
Intellectual disability, *N* (%)	351 (7.0%)	5 (0.1%)	$\chi _1^2$ = 418.2, *P* = 6.14 × 10^−93^

Premorbid cognitive ability is Z-score standardized by birth year in each sample. Educational attainment is Z-score standardized by birth year and sex in each sample. All continuous variables are described by mean and standard deviation (SD). Categorical variables are described by sample size (*N*) and percentage (%). Statistical comparisons are t-test for continuous variables and chi-square test for categorical variables. All statistical comparisons exceed Bonferroni correction (*N* = 10, *P* < 0.005).*NA*: not applicable.

### Epidemiological analyses

In the national sample, we observed strong associations between cognition and risk of SCZ in separate and joint models (Table 2A). Notably, lower premorbid cognitive ability, lower EDU, and the presence of ID were strongly associated with risk of SCZ.

**Table 2:** Epidemiological and genetic epidemiological analyses in the national sample. A. Epidemiological analyses in the Swedish national sample. B. Heritability and genetic correlations.

A						
*Trait*	*Separate model*		*Joint model 1*		*Joint model 2 (males only)*	
	*HR [95% CI]*	*P-value*	*HR [95% CI]*	*P-value*	*HR [95% CI]*	*P-value*
Premorbid cognitive ability	0.54 [0.52; 0.55]	<1 × 10^−300^	NA	NA	0.65 [0.63; 0.67]	5.87 × 10^−169^
Educational attainment	0.43 [0.42; 0.44]	<1 × 10^−300^	0.47 [0.46; 0.48]	<1 × 10^−300^	0.65 [0.63; 0.67]	1.09 × 10^−126^
Intellectual disability	13.81 [12.90; 14.79]	<1 × 10^−300^	7.54 [6.99; 8.14]	<1 × 10^−300^	12.56 [10.78; 14.65]	5.77 × 10^−229^

B				
*Trait*	*Pedigree-heritability (95% CI)*	*SNP-heritability (95% CI)*	*Pedigree-r*_*g*_ *with SCZ (95% CI)*	*SNP-r*_*g*_ *with SCZ (95% CI)*
SCZ	0.70 [0.63; 0.77]	0.24 [0.23; 0.25]	NA	NA
Premorbid cognitive ability	0.65 [0.62; 0.68]	0.19 [0.17; 0.21]	−0.11 [−0.15; −0.07]	−0.21 [−0.26; −0.16]
Educational attainment	0.37 [0.34; 0.39]	0.12 [0.12; 0.13]	0.09 [−0.04; 0.22]	0.02 [−0.01; 0.06]
Intellectual disability	0.84 [0.77; 0.91]	0.08 [0.04; 0.12]	0.50 [0.47; 0.52]	0.28 [0.15; 0.41]

Premorbid cognitive ability is Z-score standardized by birth year. Educational attainment (EDU) is Z-score standardized by birth year and sex. In Table 2A, Cox regression models are applied. Separate model tests for each cognitive trait are adjusted for sex (except for premorbid cognitive ability since it was assessed only in males), categorical birth year (1958–1962, 1963–1967, 1968–1974, 1975–1993), parental EDU (either mother’s EDU or father’s EDU if only one among them is available; if both mother’s and father’s EDU were available, take the mean), maternal age, paternal age and whether the person was born in winter (yes or no). Joint model 1 includes EDU, intellectual disability (ID) and other covariates listed as above. Joint model 2 includes premorbid cognitive ability, EDU, ID, and other covariates listed as above except for sex. All statistical comparisons exceed Bonferroni correction (*N* = 8, *P* < 0.006).

In Table 2B, for pedigree analyses, Wald confidence intervals (CI) are calculated by using the delta method. SNP-heritability and SNP-*r*_*g*_ are from the literature (SNP-*r*_*g*_ between SCZ and EDU was estimated using LDSC from the latest GWAS) [10, 22, 25, 54, 69]. Estimates and 95% CIs are shown. SNP-heritability and SNP-*r*_*g*_ in the second row refer to intelligence. SNP-heritability and SNP-*r*_*g*_ in the last row refer to severe neurodevelopmental disorders as a proxy for intellectual disability. NA: not applicable. Multiple testing correction is not applicable to this descriptive table.

### Genetic epidemiological analyses

In total, 931,744 siblings were included and results were shown in Table 2B. The pedigree-heritability for SCZ was estimated as 0.70, and the estimates of pedigree-heritability for the cognition measures were 0.37 for EDU, 0.65 for premorbid cognitive ability, and 0.84 for ID. For pedigree-genetic correlations (*r*_*g*_), SCZ had a negative pedigree-*r*_*g*_ with premorbid cognitive ability (−0.11; 95%CI: −0.15, −0.07), a positive pedigree-*r*_*g*_ with ID (0.50; 95%CI: 0.47, 0.52), and the pedigree-*r*_*g*_ with EDU was not significant (0.09; 95%CI: −0.04, 0.22). Intriguingly, these estimates of pedigree-*r*_*g*_ approximated those based on genotyping with SNP-*r*_*g*_ of SCZ with IQ (−0.21, assessed via neurocognitive tests), rare severe neurodevelopmental disorders (0.28, often comorbid including ID), and EDU (0.02) [22, 23, 54].

### Genetic burden analyses

**Fig. 1:**
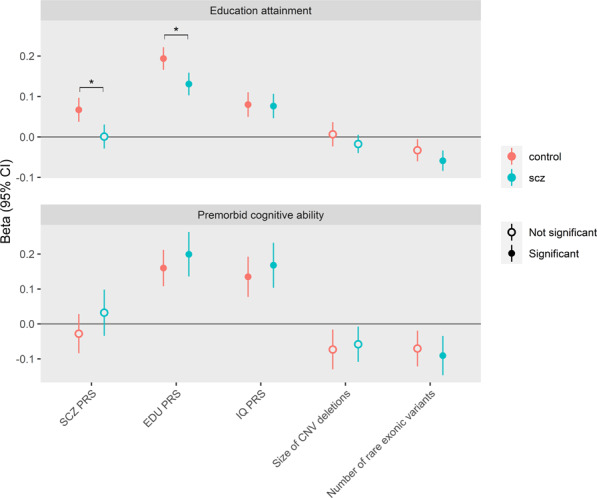
Associations between genetic burden and cognitive measures in SCZ cases and controls. Genetic profiles include: (1) polygenetic risk scores (PRS) for schizophrenia (SCZ), intelligence quotient (IQ) and educational attainment (EDU); (2) size of copy number variants (CNV) deletions in KB; and (3) rare exonic burden, measured as number of disruptive and damaging ultra-rare variants in constrained genes. Burden measures were standardized. Cognitive measures include Educational attainment (EDU) and premorbid cognitive ability (measured by intelligence quotient (IQ) scores, IQ). EDU is Z-score standardized by birth year and sex. Premorbid cognitive ability is Z-score standardized by birth year. The analysis used linear regression models including all genetic burdens above and adjusted for the first 5 ancestry principle components and genotyping waves. Beta coefficient and 95% confidence intervals (CI) are reported. Estimates past significance threshold (corrected for 20 tests, *P* < 0.0025) are marked in solid circle. Asterisk indicates significant difference between SCZ cases and controls. The data for this figure are in Table S3.

The association with SCZ was negative for PRS of IQ (OR = 0.88 (0.84–0.92), *P* = 1.38 × 10^−8^) but positive for PRS of EDU (OR = 1.08 (1.04, 1.13), *P* = 1.12 × 10^−4^). Results of associations between each burden measure and the two cognitive traits, EDU and premorbid cognitive ability, are shown in Table S2. The genetic burdens that showed significant associations were then included in the joint model, and the results are in Fig. 1 and Table S3. Since SCZ diagnosis modified the associations between EDU-PRS and EDU (Table S2), we examined the joint effect separately in cases and controls. PRS of EDU and IQ showed positive associations with cognitive traits in SCZ cases, among which the effect of EDU-PRS on EDU was lower than that in controls (0.13 vs. 0.19, P = 3.89 × 10^−4^ for test of SCZ diagnosis interaction). Rare exonic burden showed an inverse association with cognitive traits in SCZ cases (for EDU, −0.06, *P* = 4.91 × 10^−6^; for premorbid cognitive ability, −0.09, *P* = 0.002) but not in controls. SCZ-PRS had no associations with cognitive traits in SCZ cases.

### Cluster analyses in SCZ cases

We conducted unsupervised cluster analyses on 13,647 cases with complete data available for the input clustering variables. In the training set (*N* = 6823), DBSCAN clustering identified four clusters after UMAP projection (Table 3A). Cluster 1 (56.6% cases) was characterized by moderate features compared to other groups. Cluster 2 (25.5% cases) was characterized by early age at first SCZ diagnosis, lower EDU, lower parental EDU, and fewer BIP contacts. Cluster 3 (11.7% cases) was characterized by later diagnosis, higher EDU/parental EDU, and more BIP contacts. Finally, Cluster 4 (6.2% cases) SCZ cases with ID, presenting lower parental EDU. In the replication set (*N* = 6,824), using same input variables and clustering algorithm, we also identified four clusters and the individual distributions and characteristics were similar to that of the training set (Table S4). Similar patterns for other characteristics were also observed for both sets (Tables 3A, S4). For example, Cluster 2 (low EDU) had the highest proportions of males and was more likely to have long hospitalizations. Cluster 3 (high EDU) had the fewest males, lowest mortality, and was less likely to have long hospitalizations (Tables S4, S5).

**Table 3:** SCZ case characteristics across cluster groups in: A. national training set, B. genotyped subsample.

A						
*Feature*		*Cluster 1 (medium EDU)*	*Cluster 2 (low EDU)*	*Cluster 3 (high EDU)*	*Cluster 4 (ID)*	*P*
*N* (%)		3,865 (56.6%)	1,739 (25.5%)	798 (11.7%)	421 (6.2%)	–
Clustering input variables	Age at first SCZ diagnosis, mean (SD)	0.09 (0.96)	−0.23 (0.99)	0.35 (0.91)	−0.05 (1.10)	5.55×10^−48 a^
	ID, *N* (%)	0 (0%)	0 (0%)	0 (0%)	421 (100%)	–
	EDU, mean (SD)	0.12 (0.27)	−1.10 (0.27)	2.17 (0.19)	−0.70 (0.71)	<1×10^−300 a^
	Parental EDU, mean (SD)	0.03 (0.99)	−0.27 (0.90)	0.63 (1.06)	−0.43 (0.86)	3.08×10^−117 a^
	Number of BIP contacts, mean (SD)	0.01 (1.00)	−0.06 (0.76)	0.09 (1.28)	−0.03 (0.78)	0.002 ^a^
Birth year, mean (SD)		1968 (8.61)	1969 (9.03)	1969 (7.61)	1969 (8.41)	0.001 ^a^
Male sex, *N* (%)		2,444 (63.2%)	1,148 (66.0%)	408 (51.1%)	253 (60.1%)	7.04×10^−12 a^
Premorbid cognitive ability (males), mean (SD)		−0.43 (0.99)	−1.01 (0.91)	0.38 (0.90)	−1.75 (0.59)	6.38×10^−130 a^
Attempt/completed suicide, *N* (%)		497 (12.9%)	284 (16.3%)	66 (8.3%)	60 (14.3%)	4.40×10^−7 a^
Death, *N* (%)		322 (8.3%)	198 (11.4%)	28 (3.5%)	37 (8.8%)	1.53×10^−9 a^
Ever hospitalized for more than 200 days, *N* (%)		629 (16.3%)	487 (28.0%)	69 (8.6%)	76 (18.1%)	2.73×10^−36 a^
Ever use of clozapine, *N* (%)		879 (22.7%)	464 (26.7%)	141 (17.7%)	103 (24.5%)	2.61×10^−7 a^

B						
*Feature*		*Cluster 1 (medium EDU)*	*Cluster 2 (low EDU)*	*Cluster 3 (high EDU)*	*Cluster 4 (ID)*	*P*
*N* (%)		2109 (57.4%)	1064 (29.0%)	266 (7.2%)	235 (6.4%)	–
Clustering input variables	Age at first SCZ diagnosis, mean (SD)	0.07 (0.91)	−0.21 (0.93)	0.23 (0.93)	−0.14 (0.91)	1.08×10^−18^
	ID, *N* (%)	0 (0%)	0 (0%)	0 (0%)	235 (100%)	–
	EDU, mean (SD)	0.32 (0.31)	−0.98 (0.35)	2.29 (0.26)	−0.62 (0.73)	<1×10^−300^
	Parental EDU, mean (SD)	0.07 (1.01)	−0.21 (0.86)	0.66 (1.22)	−0.34 (0.84)	1.27×10^−44^
	Number of BIP contacts, mean (SD)	0.00 (0.93)	−0.06 (0.93)	0.18 (1.53)	−0.002 (0.97)	0.007
Birth Year, mean (SD)		1958 (9.41)	1957 (10.45)	1957 (9.99)	1958 (11.17)	1.86×10^−4^
Male, *N* (%)		1336 (63.3%)	701 (65.9%)	141 (53.0%)	139 (59.1%)	7.77×10^−4^
N with available data on all genetic profiles (%)		1814 (57.3%)	913 (28.9%)	234 (7.4%)	203 (6.4%)	–
PRS, mean (SD)	SCZ	0.01 (1.00)	0.01 (1.01)	−0.10 (0.96)	−0.01 (1.02)	0.44
	EDU	0.06 (1.00)	−0.19 (0.96)	0.44 (0.92)	−0.19 (1.00)	2.25×10^−20 b^
	IQ	0.03 (0.99)	−0.09 (0.99)	0.34 (1.01)	−0.21 (1.00)	6.70×10^−10 b^
	BIP	0.03 (1.01)	−0.03 (0.96)	−0.07 (1.06)	−0.04 (0.97)	0.33
CNV deletions, mean (SD)	Size of CNVs	−0.01 (0.96)	−0.03 (0.87)	−0.08 (0.58)	0.31 (1.86)	7.81×10^−5 b^
	Number of CNVs	0.00 (1.01)	−0.03 (0.96)	−0.01 (1.04)	0.14 (1.06)	0.18
	Number of known pathogenic CNVs	−0.03 (0.85)	0.01 (1.01)	−0.11 (0.03)	0.33 (2.11)	9.13×10^−6 b^
Rare exonic burden, mean (SD)	Number of disruptive or damaging ultra–rare variants in constrained genes	−0.01 (1.00)	0.05 (1.02)	−0.23 (0.86)	0.18 (1.06)	8.88×10^−5 b^

Abbreviations: *SCZ*, schizophrenia; *BIP*, bipolar disorder; *EDU*, educational attainment; *ID*, intellectual disability; *IQ*, intelligence quotient; *PRS*, polygenic risk score; *CNV*, copy number variant.

Parental EDU is either from mother or from father if only one among them is available; if both mother’s and father’s EDU are available, take the mean. In Table 3A, age at first SCZ diagnosis, EDU, parental EDU, and number of BIP contacts are regressed on birth year and sex and then take the standardized residuals within the population case cohort. Premorbid cognitive ability is Z-score standardized by birth year in the whole population cohort. The hospitalization >200 days is the median length of hospitalization for those in top decile of hospitalization. In Table 3B, except for ID, birth year and male sex, all other variables are the standardized residuals of regression models described as below: age at first SCZ diagnosis, EDU, parental EDU and number of BIP contacts are regressed on birth year and sex; PRS were regressed on the first 5 ancestry principle components (PC) and genotyping waves; CNV deletions were regressed on genotyping waves; Rare exonic burdens were regressed on PC1-PC20 estimated from whole exome sequencing and genotyping waves. Statistical comparisons are one-way ANOVA for continuous variables and chi-square test for categorical variables.

^a^Indicates results exceeding Bonferroni-corrected significance threshold (*N* = 11, *P* < 0.004) in Table 3A.

^b^Indicates results exceeding Bonferroni-corrected significance threshold (*N* = 14, *P* < 0.003) for genetic burden tests in Table 3B.

**Fig. 2:**
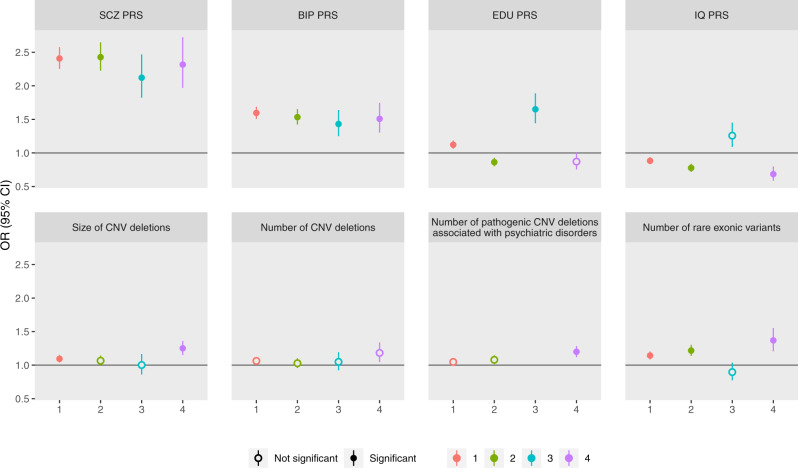
Test of genetic burden between SCZ cluster groups and controls. Genetic profiles include: (1) polygenetic risk scores (PRS) for schizophrenia (SCZ), bipolar disorder (BIP), intelligence quotient (IQ) and Educational attainment (EDU); (2) copy number variants (CNV) deletions including size of CNVs in KB, count of CNVs, and count of CNVs in pathogenic regions associated with SCZ, autism, developmental delay and intellectual disability (defined as had > 50% overlap with the region (PLINK –cnv-region-overlap 0.5)); and (3) rare exonic burden, measured as number of disruptive and damaging ultra-rare variants in constrained genes. All genetic burden measures were standardized. All analysis used logistic regression model. For PRS, analyses were adjusted for the first 5 ancestry principle components (PC) and genotyping waves. For CNV, analyses were adjusted for genotyping waves. For rare exonic burden, the analysis was adjusted for PC1-PC20 estimated from whole exome sequencing data and genotyping waves. Odds ratios (OR) and 95% confidence intervals (CI) are reported. Estimates past significance threshold (corrected for 32 tests; *P* < 0.0015) are marked in solid circle. The data for this figure are in Table S6. The test for number of known pathogenic CNVs in Cluster 3 vs. controls is not applicable because no SCZ cases in Cluster 3 had known pathogenic CNVs (empty cell).

Applying the same clustering procedures to the genotyped subsample with complete data for the input clustering variables (N = 3,674), we found that the cluster distributions and characteristics were similar to that in the populational training set (Table 3B). Moreover, the clusters differed in EDU-PRS, IQ-PRS, size of CNV deletions, number of known pathogenic CNV deletions and rare exonic burden (Table 3B). Further, the cluster 3 with high EDU had higher EDU-PRS, no lower IQ-PRS and no excess burden of rare genetic variants when compared to controls (Fig. 2, Table S6).

## Discussion

We found evidence for a robust relation between SCZ and cognitive traits by combining comprehensive national registry and directly genomic assays. In populational analyses, we confirmed that indices of worse cognition were strong risk factors for SCZ, and the pedigree-*r*_*g*_ between SCZ and cognitive traits, including EDU, ID and premorbid cognitive ability, are comparable with the SNP-*r*_*g*_ from common genetic variants. In the genotyped sample, SCZ cases were likely to have higher EDU and premorbid cognitive ability when they had higher EDU-PRS, higher IQ-PRS and less rare exonic burden. Finally, by applying an unsupervised clustering method, we found four clusters of SCZ cases characterized by EDU, age at first diagnosis, number of BIP contacts and ID in the national sample. The cases in the clusters with high EDU had less adverse outcomes including long hospitalization and death. When applying the same clustering analysis to the genetic subsample, the case cluster with high EDU presented higher PRS of EDU and no significant excess of rare genetic burdens than controls.

Multiple studies have found that lower premorbid cognitive ability is associated with multiple psychiatric disorders particularly SCZ [55, 56]. A Swedish national study found that decline in cognitive performance during the teenage years predicted psychosis in adulthood [57]. However, we cannot rule out reverse causation bias in the association between low EDU and SCZ (e.g., when onset of psychotic symptoms impaired school performance), and we note that >90% of cases achieved their highest level of education before first diagnosis of SCZ.

The pattern that the pedigree-*r*_*g*_ between SCZ and cognitive measures are comparable with previous reports and with the corresponding SNP-*r*_*g*_ presents a converging picture of the etiology for SCZ, cognitive traits and shared genetics between them [58]. We observed several interesting results. First, the SNP-heritability of severe neurodevelopmental disorders (including ID) is the smallest (0.08) [54] while its pedigree-heritability is the highest (0.84). This could be explained by current SNP genotyping arrays poorly capture rare variants with large effects which are an important contributor to severe neurodevelopmental deficits like ID. Second, we detected a significant pedigree-*r*_*g*_ between SCZ and premorbid cognitive ability in males which is in line with previous reports and with the corresponding SNP-*r*_*g*_. A Swedish twin-sibling study reported a negative genetic correlation between IQ and psychosis (−0.26), similar to the reported SNP-*r*_*g*_ (−0.21) [59]. The negative genetic correlation between premorbid cognitive ability and SCZ is also supported by our observation of positive genetic correlation between SCZ and ID. The modest negative genetic correlation (−0.11), along with a Swedish co-relative control analysis that found no attenuation in association between SCZ and intelligence in siblings, cousin pairs, and general population [18], suggests a role for non-shared environmental risk factors for lower IQ and SCZ. Third, the pedigree-*r*_*g*_ between SCZ and EDU was not significant, and was near zero SNP-*r*_*g*_ as estimated from the largest published GWAS [9, 25], despite a strong epidemiological association [24] and considerable overlap in causal variants [45].

For genetic burden analyses with cognitive traits in SCZ cases, PRS for both IQ and EDU showed positive relationship while SCZ-PRS showed no association. This is in line with a recent study that finds in SCZ cases, cognition is more strongly related with PRS that index cognitive traits in general than PRS for psychiatric disorders, suggesting the mechanisms of cognitive variation within SCZ is at least partly independent from that predisposes the illness [60]. However, unexpectedly, the EDU-PRS associated positively with SCZ risk, despite a small negative correlation between the two PRS (−0.08 in cases and −0.03 in controls). Moreover, the SCZ-PRS was positively associated with EDU in controls. Such findings were in line with some of the previous studies [61], but they are contrast to a Danish study that reported higher SCZ-PRS associated with noncompletion of primary school in SCZ noncases [62]. Recent studies have shown evidence of shared genetic loci between SCZ and EDU, and their genetic dependence possibly related to SCZ subtypes [23, 63]. Taken together with our findings of the epidemiological and pedigree analyses, it is evident that the relationship between SCZ and EDU are complex.

Previous studies on identifying SCZ subgroups have employed clustering methods based on diverse measures of cognition [64–66], and have identified subtypes with different characteristics including real-world functioning, symptom severity, clinical pattern and neurocognitive features. Here we adopted cognitive-related variables on a population-level, and finally decided a four-cluster solution. This new clustering of SCZ cases identifies individuals at different level of cognition functioning, characterized with potential different mortality rate and hospitalization length, and were genetically validated by common and rare genetic burdens. The group with high EDU tended to have higher PRS of EDU and IQ (albeit non-significant) than controls, suggesting a subgroup differed from traditional SCZ. The variables we used (i.e., age at first SCZ diagnosis, EDU, parent EDU, number of BIP contacts and ID) were common features that were frequently recorded from registers and surveys, adding the generalizability to existing SCZ cohorts and patients in clinics.

In this paper, we combined multiple interlacing approaches (national-scale epidemiology and genetic epidemiology with multiple measures of common and rare genetic variation); this is a strength of our study and uncommonly presented in a single paper. We also had several limitations. First, although our findings of no association between CNVs, SCZ-PRS and cognitive traits in SCZ cases were in line with previous studies, it could also be due to lack of power. For rare CNVs existing in a small number of carriers and explaining only a small fraction of phenotype variance, the investigation of complex cognitive traits would require extremely large dataset to achieve sufficient power [67]. Second, the Swedish National Patient Register captures only a select minority of people who might have more severe ID and comorbidities of other diseases, which limits the generalizability of the findings [68]. Third, the associations between ID and SCZ could be overestimated, because patients diagnosed with one disease are likely to be in contact with physicians and are also more likely to receive other diagnoses. Fourth, the S3 genotyped subsample may have selection bias as it requires patients to survive and have the capacity to provide informed consent. Fifth, the clustering analysis was based on complicated analytical approaches with several proxy phenotypes, and risk of overfitting cannot be ruled out. Because the health care system in Sweden is tax-funded with universal access, the generalizability of these results to other places may be limited by differences in social welfare policies, resources and practices. Replication in independent samples are warranted in the future. Last but not least, the time-varying factors that affect EDU, such as socioeconomic status and cooccurrence of diseases, were not controlled and could influence assessment of EDU. Our work could be improved via integration of longitudinal measures of cognitive function in order to better understand the association between EDU and SCZ. As these are available only for younger Swedes, further exploration will await completion of our current expansion of genotyped cases to ~12 000. Moreover, EDU is perceived as a less precise indication of cognitive abilities, which also limits the generalizability. Future studies targeting broader measures of cognition might provide a way to dissect more features of this complex disorder.

In conclusion, we sought to comprehensively understand the relation of three cognitive traits and SCZ from both epidemiological and genetic perspectives. We confirmed a negative association between premorbid cognitive ability, ID and SCZ. The relationship between EDU and SCZ are complex and warrants further examination. The data-driven clustering results suggest that combined information from a few cognition-relevant variables might usefully index the heterogeneity of SCZ, which encourages the investigation of subtype-specific mechanisms and treatments in the future.

## Supplementary information

Supplementary material

## Data Availability

Custom written R scripts used for statistical analyses can be provided upon request.
