# Supplementary material for: The impact of educational attainment, intelligence and intellectual disability on schizophrenia: a Swedish population-based register and genetic study
Source: Mol Psychiatry. 2022 Apr 5;27(5):2439–47. doi: 10.1038/s41380-022-01500-2 (PMC9135619; doi:10.1038/s41380-022-01500-2)
Supplement: Supplementary file 1 — Supplementary material [file 41380_2022_1500_MOESM1_ESM.docx]

# Supplemental Methods

## Data sources

Dates of hospitalization for inpatient treatment, specialist outpatient, deliberate self-harm, and suicide attempts (***Table S1***) were obtained from the National Patient Registry. Completed suicides were identified from the Cause of Death Registry. Information on the use of clozapine was obtained from the Prescribed Drug Register (all redeemed prescriptions in outpatient care using Anatomical Therapeutic Chemical codes, data available July 2005-December 2013). First-degree relatives were identified from the Multi-Generation Registry. The Longitudinal Integration Database for Health Insurance and Labor Market Studies provided information on years of education and sickness benefit. The Conscription Register recorded standardized IQ testing that was legally mandated for all male citizens aged 18-19 from 1901-2010. These cognition tests of logical, spatial, verbal, and technical ability were coded as a stanine sum score (i.e., standardized assuming a Gaussian distribution ranging from 1-9 with mean 5, standard deviation 2). These tests have been validated ^1^ and have high test-retest reliabilities (r=0.80-0.89 up to four years later) ^2^.

## Common variant burden (polygenetic risk scores, PRS)

Genotyping was performed in batches (Sw1-6) using different arrays at the Broad Institute; Affymetrix 5.0 (3.9%; Sw1), Affymetrix 6.0 (38.6%; Sw2-4), and Illumina OmniExpress (57.4%; Sw5-6). Genotype calling was conducted using Birdsuite (Affymetrix) or BeadStudio (Illumina). Genotype data were processed using the PGC Ricopili pipeline for quality control ^3^. Samples were excluded for missingness or high relatedness ($\hat{\pi}$ ≥ 0.2) and variants were excluded if they were not biallelic, indels, strand ambiguous, had allele frequency <0.05 or >0.95, or poor imputation (INFO score < 0.8). We applied linkage disequilibrium-based clumping to select a relatively independent set of SNPs for PRS calculation PRS (r^2^ < 0.1 in 1 Mb windows using 1000 Genomes Project European samples (phase 1 version 3) as reference, removing extended MHC region (chr6;25-34 Mb)). Using PLINK (v1.9) ^4^, PRS were calculated in the target dataset as the sum of the SNP dosages weighted by the effect from the training set across all SNPs under the pre-specified *P*-value threshold (*P* ≤ 0.05). PRS were standardized in each genotyping wave to account for variation in the numbers of SNPs used across different genotyping chips.

## Rare copy number variant (CNV) burden

CNV processing details are provided previously ^5^. Briefly, CNVs were detected using Birdseye ^6^. We removed low-confidence CNVs with confidence scores < 10, < 10 probes, or < 10 kb in length followed by removal of CNVs with > 50% reciprocal overlap with large genomic gaps (e.g., centromeres) or regions subject to rearrangement in white blood cells. We annealed adjoining CNVs that appeared to be artificially split by recursively joining CNVs if the called region was ≥ 80% of the entire region to be joined. The largest CNVs (≥5 Mb) and chrX CNVs were visually inspected and those of low confidence were removed. We excluded subjects with excessive noise or CNV calls scattered across many chromosomes (≥ 40 segments or total length ≥ 6 Mb).

## Rare exonic burden

Full details of whole exome sequencing details are reported elsewhere ^7, 8^. Briefly, we used Agilent SureSelect Human All Exon Kits (v1 and v2) and sequenced on Illumina GAII, HiSeq 2000, or HiSeq 2500 instruments (76 bp paired-end sequencing). Sequencing reads were aligned to the GRCh37 human genome reference and processed using GATK with genotype calls generated using GATK Haplotype Caller. Variants filtered out by the GATK Variant Quality Score Recalibration tool were excluded. Genotypes of sites with <10x coverage were set to missing. The call set used was also included in the Exome Aggregation Consortium study ^9^.

Details of variant annotation are described elsewhere ^7^. Briefly, all genotyped variants were annotated with SnpEff ^10^ using Ensembl gene models from database GRCh37 and were further annotated with SnpSift ^11^ using annotations from database dbNSFP.^12, 13^ Among which, *damaging variants* were missense variants predicted to compromise protein function by an algorithm (SIFT, PolyPhen-2, LRT, Mutation Taster, Mutation Assessor or PROVEAN), in-frame indels or variants affecting protein-protein-contact domains; *disruptive variants*, including frame shift, nonsense, splice-site and, very rarely, read-through variants, were those truncating the encoded protein in a way that was readily classified as loss of function or as triggering nonsense-mediated decay. We further identified those disruptive and damaging ultra-rare variants that are in subsets of human genes that have been previously identified as 'missense-constrained' (based on a lack of functional coding variation in controls) or 'loss-of-function intolerant' (based on a smaller-than-expected number of loss-of-function mutations in population-scale data) ^14, 15^.

## Assumptions of pedigree-heritability analyses of schizophrenia (SCZ) and cognitive ability

As in classical twin models, we assumed that monozygotic twins shared all of their co-segregating genes while dizygotic twins and non-twin full-siblings shared 50% of their co-segregating genes and half-siblings 25% of their co-segregating genes. We assumed that all twins and non-twin full siblings shared their childhood family environments, and we fixed the correlation to 1 for maternal half-siblings and 0 for paternal half-siblings, consistent with previous Swedish quantitative genetic studies ^16-19^. Finally, we assumed that all twin and sibling pairs were uncorrelated in regard to their unique environmental influences.

***Table S1. Diagnostic codes from the World Health Organization International Classification of Diseases (ICD) used in Sweden***

| *Psychiatric disorder* | *ICD-8* | *ICD-9* | *ICD-10* |
| --- | --- | --- | --- |
| Bipolar disorder | 296.1, 296.3, 296.8 | 296, 269A, 269C, 296D, 296E, 296F, 296G, 269H, 269W, 269X | F30, F301, F302, F308, F309, F31, F31.1-F31.9 |
| Intellectual disability | 310-315 | 317-319 | F70-F73, F78-F79 |
| Major depressive disorder | 300,4 | 296.3, 311 | F32, F32.0-F32.3, F32.8, F32.9, F33, F33.0-F33.4, F33.8, F33.9, F34.8, F34.9, F38, F38.0, F38.1-F38.8, F39 |
| Schizoaffective disorder | 295.7 | 295.7 | F25, F25.0-F25.2, F25.8, F25.9, F23.1, F23.2 |
| Schizophrenia * | 295.0-295.4, 295.6-295.9 | 295.0-295.4, 295.6-295.9 | F20, F20.0-F20.6, F20.8, F20.9 |
| Substance use disorders | 303, 303.1, 303.2, 303.9, 304, 304.1-304.9 | 303, 303A, 303X, 304, 304A-304H, 304W, 304X, 305A, 305X | F10, F10.0-F10.9, F11, F11.0-F11.9, F19, F19.0-F19.9 |
| Suicide | E950-E959 | E95A-E95H, E95W, E95X | X60-X84 |

* We excluded cases with a plausible alternative primary diagnoses; (a) ≤5 treatment contacts for schizophrenia but ≥5 treatment contacts for bipolar disorder or (b) ≥50 treatment contacts for substance use disorder.

***Table S2. Associations between genetic burden and cognitive traits in combined sample (separate model)***

|  | | Educational attainment | Premorbid cognitive ability |
| --- | --- | --- | --- |
| ***Polygenetic risk score (PRS)*** | | Beta (95% CI); P | Beta (95% CI); P |
| EDU | | 0.29 (0.23,0.34); 3.47×10^-22^ **‡** | 0.23 (0.19,0.26); 5.82×10^-30^ **‡** |
| IQ | | 0.13 (0.11,0.16); 3.86×10^-37^ **‡** | 0.21 (0.17,0.25); 5.96×10^-24^ **‡** |
| BIP | | 0.02 (-0.002, 0.04); 0.07 | -0.02 (-0.06,0.02); 0.37 |
| SCZ | | 0.11 (0.05,0.18); 5.36×10^-4^ **‡** | -0.03 (-0.08,0.01); 0.15 |
| Interaction EDU-PRS and SCZ diagnosis | | -0.06 (-0.10, -0.03); 0.001 **‡** | - |
| Interaction SCZ-PRS and SCZ diagnosis | | -0.06 (-0.10, -0.02); 0.007 | - |
| ***Rare copy number variation (CNV)*** | | | |
| Deletions | Size of CNVs | -0.01 (-0.03, 0.01); 0.36 | -0.06 (-0.10, -0.03); 0.001 **‡** |
|  | Numer of CNVs | -0.01 (-0.03, 0.01); 0.31 | -0.03 (-0.07,0.005); 0.09 |
|  | Number of known pathogenic CNVs | -0.02 (-0.04, 0.001); 0.07 | -0.04 (-0.08,0.003); 0.07 |
| Duplications | Size of CNVs | 0.01 (-0.01, 0.03); 0.30 | 0.00 (-0.04,0.04); 1.00 |
|  | Number of CNVs | 0.001 (-0.02,0.02); 0.94 | 0.001 (-0.04,0.04); 0.96 |
|  | Number of known pathogenic CNVs | -0.01 (-0.03,0.01); 0.41 | -0.03 (-0.07,0.01); 0.20 |
| ***Rare exonic burden*** | | | |
| Disruptive/damaging ultra-rare variation in constrained genes | | -0.05 (-0.07, -0.03); 3.98×10^-7^ **‡** | -0.08 (-0.12, -0.05); 2.14×10^-5^ **‡** |

Abbreviations; EDU, educational attainment; SCZ, schizophrenia; IQ, intelligence quotient. PRS models adjusted for case/control status, ancestry PC1-PC5 and genotyping waves. CNV models adjusted for case/control status and genotyping waves. Rare exonic models adjusted for case/control status, PC1-PC20 estimated from whole exome sequencing and genotyping waves. When testing PRS of EDU and SCZ for the outcome of EDU, the interaction between the PRSs and SCZ diagnosis was included in the model. A total of 11 genetic burden measures were tested (4 PRS, 6 CNV, 1 rare exonic burden) for two cognitive traits. **‡** Indicates results exceeding Bonferroni-corrected significance threshold (N=22, P<0.002)

***Table S3. Associations between genetic burden and cognitive traits in SCZ cases and controls (joint model)***

|  | Education attainment | | | Premorbid cognitive ability | |
| --- | --- | --- | --- | --- | --- |
|  | Beta (95% CI); P | | | Beta (95% CI); P | |
|  | SCZ | | Control | SCZ | Control |
| ***Polygenic risk score (PRS)*** | | | | | |
| EDU PRS | 0.13 (0.10,0.16); 1.07×10^-19^ **‡** | | 0.19 (0.17,0.22); 2.23×10^-41^ **‡** | 0.20 (0.14, 0.26); 1.12×10^-9^ **‡** | 0.16 (0.11,0.21); 2.36×10^-9^ **‡** |
| IQ PRS | 0.08 (0.05,0.11); 7.58×10^-7^ **‡** | | 0.08 (0.05,0.11); 3.02×10^-7^ **‡** | 0.17 (0.10,0.23); 3.81×10^-7^ **‡** | 0.135 (0.08,0.19); 4.84×10^-6^ **‡** |
| SCZ PRS | 0.00 (-0.03,0.03); 0.96 | | 0.07 (0.04,0.10); 1.00×10^-5^ **‡** | 0.03 (-0.03,0.10); 0.34 | -0.03 (-0.08,0.03); 0.33 |
| ***Rare copy number (CNV)*** | | | | | |
| Size of CNV deletions | -0.02 (-0.04,0.01); 0.13 | | 0.01 (-0.02,0.04); 0.67 | -0.06 (-0.11, -0.01); 0.02 | -0.07 (-0.13, -0.02); 0.01 |
| ***Rare exonic burden*** | | | | | |
| Disruptive/damaging ultra-rare variation in constrained genes | | -0.06 (-0.08, -0.03); 4.91×10^-6^ **‡** | -0.03 (-0.06, -0.01); 0.02 | -0.09 (-0.15, -0.03); 0.002 **‡** | -0.07 (-0.12, -0.02); 0.007 |

Abbreviations; EDU, educational attainment; SCZ, schizophrenia; IQ, intelligence quotient. Joint model adjusts for ancestry PC1-PC5, genotyping waves, and PC1-PC20 estimated from whole exome sequencing. A total of 5 genetic burden measures were tested (3 PRS, 1 CNV, 1 rare exonic burden) in cases and controls separately for EDU and premorbid cognitive ability. The P-value was corrected for 5x2x2=20 tests. **‡** Indicates results exceeding Bonferroni-corrected significance threshold (N=20, P< 0.0025).

***Table S4. SCZ case characteristics across cluster groups in national replication set***

| *Feature* | | *Cluster 1* | *Cluster 2* | *Cluster 3* | *Cluster 4* | *P* |
| --- | --- | --- | --- | --- | --- | --- |
| N (%) | | 3,833 (56.2%) | 1,778 (26.1%) | 833 (12.2%) | 380 (5.6%) | - |
| Input clustering variables | Age at first SCZ diagnosis, mean (SD) | 0.07 (0.95) | -0.22 (0.99) | 0.37 (0.87) | -0.07 (1.13) | 5.45×10^-50^ **‡** |
|  | ID, N (%) | 0 (0%) | 0 (0%) | 0 (0%) | 380 (100%) | - |
|  | EDU, mean (SD) | 0.12 (0.28) | -1.09 (0.24) | 2.19 (0.23) | -0.69 (0.68) | <1×10^-300^ **‡** |
|  | Parental EDU, mean (SD) | 0.04 (0.99) | -0.23 (0.88) | 0.60 (1.02) | -0.44 (0.88) | 6.23×10^-108^ **‡** |
|  | Number of BIP contacts, mean (SD) | 0.01 (0.96) | -0.05 (0.91) | 0.15 (1.39) | 0.00 (1.54) | 8.73×10^-5^ **‡** |
| Birth Year, mean (SD) | | 1969 (8.49) | 1969 (9.00) | 1969 (7.61) | 1969 (8.36) | 0.002 **‡** |
| Male sex, N (%) | | 2,365 (61.7%) | 1,166 (65.6%) | 428 (51.4%) | 203 (53.4%) | 1.55×10^-12^ **‡** |
| Premorbid cognitive ability (males), mean (SD) | | -0.45 (1.02) | -1.08 (0.88) | 0.44 (0.90) | -1.80 (0.57) | 1.20×10^-141^ **‡** |
| Attempt/completed suicide, N (%) | | 469 (12.2%) | 273 (15.4%) | 79 (9.5%) | 53 (13.9%) | 1.59×10^-4^ **‡** |
| Death, N (%) | | 288 (7.5%) | 176 (9.9%) | 38 (4.6%) | 27 (7.1%) | 2.70×10^-5^ **‡** |
| Ever hospitalized for more than 200 days, N (%) | | 663 (17.3%) | 459 (25.8%) | 74 (8.9%) | 86 (22.6%) | 6.41×10^-26^ **‡** |
| Use of clozapine, N (%) | | 875 (22.8%) | 472 (26.5%) | 130 (15.6%) | 94 (24.7%) | 1.47×10^-9^ **‡** |

Abbreviations; SCZ, schizophrenia; BIP, bipolar disorder; EDU, educational attainment; ID, intellectual disability. Parental EDU is either from mother or from father if only one among them is available; if both mother’s and father’s EDU are available, take the mean. Age at first SCZ diagnosis, EDU, parental EDU, and number of BIP contacts are regressed on birth year and sex and then take the standardized residuals within the population case cohort. Premorbid cognitive ability is Z-score standardized by birth year in the whole population cohort (as defined previously). The hospitalization >200 days is the median length of hospitalization for those in top decile of hospitalization. Statistical comparisons are one-way ANOVA for continuous variables and chi-square test for categorical variables. **‡** Indicates results exceeding Bonferroni-corrected significance threshold (N=11, P < 0.004).

***Table S5. Multivariable Cox regression analyses of adverse outcomes across cluster groups in Swedish national schizophrenia cases (combining training set and replication set)***

| *Outcome* | *Cluster*  *(1 as ref)* | *Hazard Ratio* | *Lower 95% CI* | *Upper 95% CI* | *P* |
| --- | --- | --- | --- | --- | --- |
| Suicide-related event | 2 (low EDU) | 1.00 | 0.89 | 1.11 | 0.93 |
|  | 3 (high EDU) | 0.83 | 0.68 | 1.00 | 0.05 |
|  | 4 (ID) | 1.05 | 0.86 | 1.28 | 0.65 |
| First hospitalization  >200 days | 2 (low EDU) | 1.36 | 1.24 | 1.48 | 1.51×10^-1^^1^ **‡** |
|  | 3 (high EDU) | 0.67 | 0.56 | 0.80 | 8.60×10^-6^ **‡** |
|  | 4 (ID) | 1.06 | 0.89 | 1.25 | 0.52 |
| Death | 2 (low EDU) | 1.16 | 1.01 | 1.32 | 0.03 |
|  | 3 (high EDU) | 0.60 | 0.46 | 0.78 | 1.77×10^-4^ **‡** |
|  | 4 (ID) | 1.03 | 0.79 | 1.33 | 0.84 |

Abbreviation; EDU, educational attainment; ID, intellectual disability. Cox proportional hazards regression models were used, with Cluster 1 (medium EDU) as the reference group. All models were adjusted for age at first SCZ diagnosis, substance use disorders (SUD), anxiety disorders, major depression and categorical birth year (1958-1962, 1963-1967, 1968-1974, 1975-1995, grouped by quartiles of the birth year distribution). The test for suicide-related events was additionally adjusted for history of suicide-related events before SCZ diagnosis. For first hospitalization >200 days, the test was additionally adjusted for receiving sickness pension (yes or no). **‡** Indicates results exceeding Bonferroni-corrected significance threshold (N=9, P < 0.005).

***Table S6. Test of genetic burden between SCZ cluster groups and controls***

| ***Polygenetic risk score (PRS)*** | OR (95% CI); P | | | |
| --- | --- | --- | --- | --- |
|  | *Cluster 1*  *(medium EDU)* | *Cluster 2*  *(low EDU)* | *Cluster 3*  *(high EDU)* | *Cluster 4*  *(ID)* |
| EDU PRS | 1.12 (1.06,1.18); 3.03×10^-5^ **‡** | 0.86 (0.80,0.93); 5.66×10^-5^ **‡** | 1.65 (1.44,1.89); 3.07×10^-13^ **‡** | 0.87 (0.75,1.00); 0.06 |
| IQ PRS | 0.88 (0.83,0.94); 2.75×10^-5^ **‡** | 0.78 (0.72,0.84); 1.74×10^-10^ **‡** | 1.26 (1.09,1.45); 0.002 | 0.68 (0.59,0.80); 1.71×10^-6^ **‡** |
| SCZ PRS | 2.41 (2.25,2.58); 1.16×10^-144^ **‡** | 2.43 (2.23,2.65); 9.26×10^-90^ **‡** | 2.12 (1.82,2.47); 8.55×10^-23^ **‡** | 2.32 (1.97,2.72); 1.44×10^-24^ **‡** |
| BIP PRS | 1.60 (1.51,1.69); 6.18×10^-59^ **‡** | 1.53 (1.42,1.65); 3.45×10^-29^ **‡** | 1.43 (1.25,1.64); 2.05×10^-7^ **‡** | 1.51 (1.30,1.75); 3.90×10^-8^ **‡** |
| ***Rare CNV deletions*** | | | | |
| Size of CNVs | 1.09 (1.04,1.15); 2.63×10^-4^ **‡** | 1.07 (0.99,1.14); 0.04 | 1.00 (0.86,1.16); 0.49 | 1.25 (1.15,1.36); 7.15×10^-8^ **‡** |
| Number of CNVs | 1.06 (1.01,1.12); 0.01 | 1.03 (0.96,1.10); 0.22 | 1.05 (0.92,1.19); 0.23 | 1.18 (1.04,1.34); 0.004 |
| Number of known pathogenic CNVs | 1.05 (0.99,1.11); 0.04 | 1.08 (1.02,1.15); 0.01 | NA | 1.20 (1.12,1.28); 7.31×10^-8^ **‡** |
| ***Rare exonic burden*** | | | | |
| Disruptive/damaging ultra-rare variants in constrained genes | 1.14 (1.08,1.20); 3.31×10^-7^ **‡** | 1.22 (1.14,1.30); 5.34×10^-9^ **‡** | 0.90 (0.77,1.04); 0.07 | 1.37 (1.21,1.55); 4.96×10^-7^ **‡** |

Abbreviations; EDU, educational attainment; ID, intellectual disability; SCZ, schizophrenia; BIP, bipolar disorder; IQ, intelligence quotient; CNV, copy number variant. PRS models adjust for ancestry PC1-PC5 and genotyping waves. Rare CNV models adjust for genotyping waves. Rare exonic model adjusts for PC1-PC20 estimated from whole exome sequencing and genotyping waves. Tests for SCZ-PRS, all CNV deletions and ultra-rare variants were one-sided assuming higher burden in cases; tests for PRSs of EDU, IQ and BIP are two-sided. **‡** Indicates results exceeding Bonferroni-corrected significance threshold (N=32, P < 0.0015). The test for number of known pathogenic CNVs in Cluster 3 vs. controls is not applicable because no SCZ cases in Cluster 3 had known pathogenic CNVs (empty cell).

# References

1. Carlsted B, Mårdberg B. Construct validity of the Swedish enlistment battery. *Scandinavian Journal of Psychology* 1993; **34:** 353-362.

2. Ross A. De värnpliktigas prestationsförmåga vid inskrivningsprövningar i Sverige 1969–1979 [The conscripts’ capacity at enlistment testing in Sweden 1969–1979]. *University of Umeå, , Pedagogiska Institutionen, Umeå* 1988.

3. Lam M, Awasthi S, Watson HJ, Goldstein J, Panagiotaropoulou G, Trubetskoy V *et al.* RICOPILI: Rapid Imputation for COnsortias PIpeLIne. *Bioinformatics* 2020; **36**(3)**:** 930-933.

4. Chang CC, Chow CC, Tellier LC, Vattikuti S, Purcell SM, Lee JJ. Second-generation PLINK: rising to the challenge of larger and richer datasets. *Gigascience* 2015; **4:** 7.

5. Szatkiewicz J, O'Dushlaine C, Chen G, Chambert K, Moran J, Neale B *et al.* Copy number variation in schizophrenia in Sweden. *Molecular Psychiatry* 2014; **19:** 762-773.

6. Korn JM, Kuruvilla FG, McCarroll SA, Wysoker A, Nemesh J, Cawley S *et al.* Integrated genotype calling and association analysis of SNPs, common copy number polymorphisms and rare CNVs. *Nat Genet* 2008; **40**(10)**:** 1253-1260.

7. Genovese G, Fromer M, Stahl EA, Ruderfer DM, Chambert K, Landen M *et al.* Increased burden of ultra-rare protein-altering variants among 4,877 individuals with schizophrenia. *Nature Neuroscience* 2016; **19**(11)**:** 1433-1441.

8. Purcell SM, Moran JL, Fromer M, Ruderfer D, Solovieff N, Roussos P *et al.* A polygenic burden of rare disruptive mutations in schizophrenia. *Nature* 2014; **506:** 185-190.

9. Exome Aggregation Consortium, Lek M, Karczewski K, Minikel E, Samocha K, Banks E *et al.* Analysis of protein-coding genetic variation in 60,706 humans. *Nature* 2016; **536**(7616)**:** 285-291.

10. Cingolani P, Platts A, Wang le L, Coon M, Nguyen T, Wang L *et al.* A program for annotating and predicting the effects of single nucleotide polymorphisms, SnpEff: SNPs in the genome of Drosophila melanogaster strain w1118; iso-2; iso-3. *Fly (Austin)* 2012; **6**(2)**:** 80-92.

11. Abe C, Petrovic P, Ossler W, Thompson WH, Liberg B, Song J *et al.* Genetic risk for bipolar disorder and schizophrenia predicts structure and function of the ventromedial prefrontal cortex. *Journal of psychiatry & neuroscience : JPN* 2021; **46**(4)**:** E441-E450.

12. Liu X, Jian X, Boerwinkle E. dbNSFP: a lightweight database of human nonsynonymous SNPs and their functional predictions. *Hum Mutat* 2011; **32**(8)**:** 894-899.

13. Liu X, Wu C, Li C, Boerwinkle E. dbNSFP v3.0: A One-Stop Database of Functional Predictions and Annotations for Human Nonsynonymous and Splice-Site SNVs. *Hum Mutat* 2016; **37**(3)**:** 235-241.

14. Pinto D, Delaby E, Merico D, Barbosa M, Merikangas A, Klei L *et al.* Convergence of genes and cellular pathways dysregulated in autism spectrum disorders. *Am J Hum Genet* 2014; **94**(5)**:** 677-694.

15. Iossifov I, O'Roak BJ, Sanders SJ, Ronemus M, Krumm N, Levy D *et al.* The contribution of de novo coding mutations to autism spectrum disorder. *Nature* 2014; **515**(7526)**:** 216-221.

16. Sandin S, Lichtenstein P, Kuja-Halkola R, Larsson H, Hultman CM, Reichenberg A. The familial risk of autism. *JAMA* 2014; **311**(17)**:** 1770-1777.

17. Viktorin A, Meltzer-Brody S, Kuja-Halkola R, Sullivan PF, Landen M, Lichtenstein P *et al.* Heritability of Perinatal Depression and Genetic Overlap With Nonperinatal Depression. *Am J Psychiatry* 2016; **173**(2)**:** 158-165.

18. Yao S, Larsson H, Norring C, Birgegard A, Lichtenstein P, D'Onofrio BM *et al.* Genetic and environmental contributions to diagnostic fluctuation in anorexia nervosa and bulimia nervosa. *Psychol Med* 2019**:** 1-8.

19. Skoglund C, Tiger A, Ruck C, Petrovic P, Asherson P, Hellner C *et al.* Familial risk and heritability of diagnosed borderline personality disorder: a register study of the Swedish population. *Mol Psychiatry* 2019.
